# Immunoprotective test and whole-genome sequencing analysis of the attenuated S02 strain of *Streptococcus iniae*

**DOI:** 10.3389/fmicb.2025.1550544

**Published:** 2025-06-03

**Authors:** Dandan Yi, Aiying Lei, Yu Liu, Guixiang Tong, Ting Huang, Chenyu Quan, Ming Chen, Liping Li

**Affiliations:** ^1^College of Animal Science and Technology, Guangxi University, Nanning, Guangxi, China; ^2^China (Guang Xi)-ASEAN Key Laboratory of Comprehensive Exploitation and Utilization of Aquatic Germplasm Resources, Ministry of Agriculture and Rural Affairs, Nanning, China; ^3^Guangxi Key Laboratory of Aquatic Genetic Breeding and Healthy Aquaculture; Guangxi Academy of Fishery Sciences, Nanning, China; ^4^Guangxi Veterinary Research Institute, Nanning, China; ^5^Key Laboratory of Veterinary Biotechnology of Guangxi, Nanning, China; ^6^Key Laboratory of China (Guangxi)-ASEAN Cross-border Animal Disease Prevention and Control, Ministry of Agriculture and Rural Affairs, Nanning, China

**Keywords:** *Streptococcus iniae*, tilapia, attenuated vaccine, immune protection, whole genome sequencing

## Abstract

**Background:**

*Streptococcus iniae* is one of the most serious diseases threatening tilapia aquaculture, causing huge economic losses every year. Injectable attenuated vaccines are still the best choice for preventing streptococcal diseases affecting the tilapia.

**Objective:**

This study evaluated the safety, stability, immunogenicity, antibody production time, and immune dose of the attenuated S02 strain of *S. iniae* and comprehensively analyzed the possible mechanisms of its attenuated virulence at the whole-genome level.

**Results:**

After detoxification, the S02 strain completely loses its pathogenicity to tilapia and has good immunogenicity. The results of the backpropagation safety test showed that the S02 strain did not cause disease or death to tilapia after continuous passage for 50 generations. AfterS02 was injected, the immunoglobulin M (IgM) level in the serum was significantly higher than that in the GX005 infection group within 28 days and reached its peak at 14 days. An intraperitoneal injection of 10^9^ CFUs/mL of S02 at a dose of 0.2 mL per fish had the best relative protection rate of 92.58%. The whole-genome sequencing results showed that the S02 strain had two large 0.2 Mbp segments of inversion compared to its parent virulence strain GX005, encoding 372 genes, including the virulence genes of the GNAT family N-acetyltransferase and the hyaluronic acid lyase genes of the hysA, hylA, and hylB, which are related to virulence factors.

**Conclusion:**

This study provides theoretical data support for the prevention and control of the *S. iniae* infection in tilapia. The abnormal expression of important virulence genes GNAT family N-acetyltransferase and hyaluronic acid lyase genes hysA, hylA, and hylB caused by the inversion and translocation of large fragments could be the main mechanism for their attenuated virulence. This study provided theoretical support for the prevention and treatment of *S. iniae* infection in tilapia and the application of S02-attenuated vaccine.

## Graphical abstract

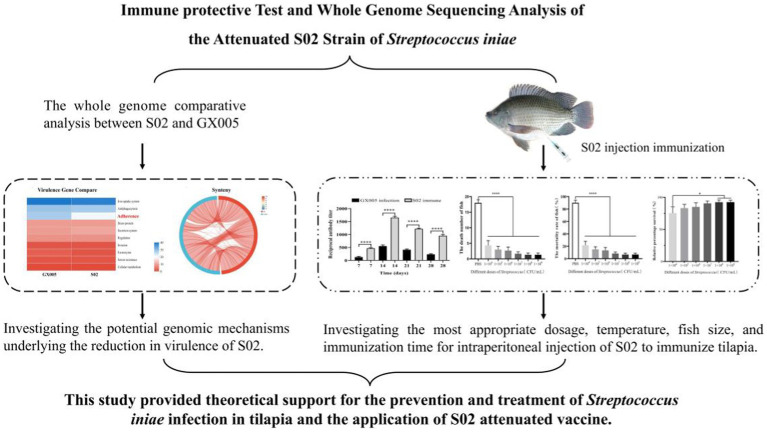

## 1 Introduction

*Streptococcus*, as one of the most serious aquatic pathogens causing invasive diseases in fish, is prevalent worldwide (Goh et al., 1998). *Streptococcus iniae* is an emerging zoonotic pathogen (Baiano and Barnes, 2009) that may cause bacteremia, cellulitis, and meningitis in humans and fish (Wang et al., 2015; El Aamri et al., 2015). *S. iniae* can spread from wild fish to farmed fish and the infection it causes poses an increasing threat to aquaculture (Zlotkin et al., 1998). Meanwhile, an *S. iniae* infection can cause bacterial hemolytic cellulitis and myelitis (Lau et al., 2003), posing a serious threat to aquaculture and human health worldwide (Yin et al., 2022). The current prevention and control of *S. iniae* mainly relies on antibiotics. Unreasonable use or a long-term application of antibiotics leads to frequent problems such as drug residues in fish bodies, food safety hazards, and pollution of water environments, making the prevention and control of *S. iniae* increasingly difficult (Schar et al., 2021).

According to data published by the Food and Agriculture Organization of the United Nations (FAO), the current proportion of aquaculture production within the global seafood output is approximately equivalent to that of wild fisheries. The notable expansion observed in the aquaculture market suggests a trend toward aquaculture surpassing wild fisheries as the primary source of fish in the forthcoming years (FAO, 2021). Tilapia, considered one of the most versatile fish species (Bovolenta et al., 2020), is the third most cultivated fish worldwide. Streptococcal disease typically occurs in intensive tilapia farming systems, posing significant risks and losses to its farming and production (Liu et al., 2016). The streptococcal infection can result in high mortality rates within a period of 3–7 days, with fish experiencing chronic outbreaks that may persist for several weeks and continue to exhibit mortality. Elevated water temperatures prevalent in tropical and subtropical regions provide particularly favorable conditions for the proliferation of this Gram-positive bacterium (Awate et al., 2023). Therefore, overcoming the Streptococcal disease of tilapia is urgent. In addition to antibiotic prevention and treatment, new strategies have emerged, such as bacteriophage endolysin therapy (Deshotel et al., 2024), *Probiotic Bacillus safensis NPUST1* (Wu et al., 2021), deep-sea fungus *Simplicidium oblivatum EIODSF 020* (Liang et al., 2024), natural drugs (Rangel-López et al., 2020; Oh et al., 2022; Abdelkhalek et al., 2020), and the development of attenuated vaccines (Wang et al., 2014), to address this challenge of Streptococcal infection. Compared to other methods, vaccines are the most efficient and beneficial form of protection. Vaccines can directly stimulate the body to produce an immune response, producing a large number of antibodies to fight against streptococcal infections. In recent years, injectable attenuated vaccines have the advantages of high efficiency, accuracy, and wide range. However, there are certain problems in the development of intraperitoneal attenuated vaccines targeting streptococci, which limit their practice and application in the aquatic industry, such as high toxicity recovery risk, unclear protection period, short protection period, and poor protection ability (Heckman et al., 2022). At present, one of the methods to obtain attenuated strains is through low-dose antibiotic screening of the antibiotic-resistant strains (Jiang et al., 2016), but this method carries the risk of virulence recovery. Another approach is to use techniques such as random transposon mutagenesis and gene recombination (Liu et al., 2019; Ganeshalingam et al., 2022). In the latter method, random transposon mutation is the most common because it has the advantage of simple operation.

Whole-genome sequencing has gained widespread application in visualizing the high-resolution characteristics of bacterial pathogens (Cao et al., 2022). The genomic information that has been revealed will facilitate the identification and understanding of potential virulence genes and candidate immunogens within the *Streptococcus* species. This knowledge will subsequently be harnessed for the development of efficacious vaccines against streptococcal diseases. Recently, the genomic information from many pathogenic *S. iniae* has been encoded, which confirms the genomic diversity of this species. For instance, *S. iniae* strain 89,353 is a virulent isolate obtained from tilapia that exhibit disease symptoms in Taiwan. The complete genome sequence of *S. iniae* strain 89,353 comprises 2,098,647 base pairs (bp) (Gong et al., 2017). Seven novel strains of the bacterial pathogen *S. iniae* were isolated from commercial fish farms located in Singapore and Australia. Genomic analysis revealed that these strains exhibit a close phylogenetic relationship, yet they represent distinct lineages within the *S. iniae* species. Inoculation with the inactivated strain p3sab was found to confer cross-protection against infection by various strains of *S. iniae*, identified in both Singapore and Australia, *in vivo* in *Lates calcifer* (Awate et al., 2023). The pathogenic strain of *S. iniae*, designated as 2022si08, was isolated and identified from diseased *Tachysurus fulvidraco* (yellow catfish) at a farm located in Hubei Province, China. This particular strain was responsible for a significant mortality rate among the yellow catfish population. The complete genome of *S. iniae* strain 2022SI08 was sequenced and predicted to consist of a 1,776,777 bp monocyclic chromosome, with a guanine–cytosine (GC) content of 37.14%. Genomic sequence analysis indicated that strain 2022si08 harbored 204 virulence genes and 127 antibiotic resistance genes (Xu et al., 2024).

We previously prepared YM011 from its parent virulence strain GX005 through 800 consecutive *in vitro* passages (Liu et al., 2020). Similarly, we isolated the attenuated SO2 strain through successive passages. We evaluated the safety, stability, immunogenicity, antibody production time, immune dose, and immune protection period of S02 and performed a whole-genome comparative sequencing of GX005 and S02 strains, aiming to provide a genomic basis for the virulence attenuation mechanism of the fish-derived attenuated streptococcal vaccine strain S02 and provide a theoretical support for its clinical application.

## 2 Methods

### 2.1 Strains and experimental fish

*S. iniae* GX005 was isolated from a tilapia in Yongjiang, Nanning, Guangxi in 2006. The fish exhibited typical symptoms of meningitis. Our previous research indicated that this strain had good immunogenicity and was a good vaccine candidate strain (Ganeshalingam et al., 2022).

The tilapia used in this study were provided by a national tilapia breeding farm and were confirmed to be pathogen-free. Prior to the formal experiment, tilapia was temporarily kept in an 800-L thickened plastic water tank for 2 weeks, at a water temperature of 30 ± 0.3°C and in 4 g/L of dissolved oxygen. The experimental group fish were raised in a 40 L plastic tank, each with an independent circulation system and an external biofilter system (China Huasheng), and were fed with a puffed formula feed typically used for tilapia (China Nanning Tongwei Biotechnology Co., Ltd) twice a day. The study was conducted in accordance with the recommendations of the Academy of Animal Research Guidelines and approved by the Animal Ethics Committee of Guangxi Academy of Fisheries (protocol number: GAFS2023001) to comply with the ethical aspects of the study involving experimental animals. All animal experiments involving fish protocols and procedures were performed in accordance with the protocols for the care of laboratory animals stipulated by the Ministry of Science and Technology People’s Republic of China. The fish were euthanized using anesthesia to ensure minimal suffering.

### 2.2 Generation and attenuation of virulence in strain GX005

Strain GX005 was retrieved from a −80°C freezer and subsequently streaked onto a 5% blood agar plate. Following incubation at 28°C for 24 h, a single colony was isolated and inoculated in 10 mL of tryptone soy broth (TSB) medium. This culture was then incubated with shaking at 28°C for 12 h. Subsequently, 2.0 mL of the bacterial suspension was transferred into a 10 mL of a fresh TSB medium, and the process of shaking incubation was repeated for 24 h. This serial passage protocol was maintained, with each passage occurring every 24 h.

To attenuate the virulence of GX005 in tilapia, experimental fish with a mean weight of 60 ± 10.19 g were selected. Every 60 generations, an infection test was conducted to assess the pathogenicity of the serially passaged strains toward tilapia. This was continued until a concentration of 1.0 × 10^9^ CFUs/mL, administered intraperitoneally (*n* = 20), which failed to induce disease or mortality.

A safety test further confirmed that after 300 consecutive generations of passage, no disease or mortality was observed in the tilapia. The resultant attenuated strain was designated as S02.

### 2.3 Toxicity comparison test between S02 and GX005

The attenuated S02 strain and the virulent GX005 strain were removed from the refrigerator maintained at −80°C, streaked and inoculated onto a blood plate, and incubated at 28°C for 24 h. A single colony was selected and inoculated in 100 mL of TSB medium. This colony in the TSB medium was incubated at 28°C with low-speed oscillation for 24 h, and the bacterial concentration was calculated by counting the colonies on the blood plate.

To investigate the toxicity of S02 and GX005 in tilapia, experimental fish of 60 ± 10.19 g were selected. The toxicity of S02 and GX005 was compared by intraperitoneal injection. The experiment was divided into three groups: GX005 group (*n* = 120) and S02 injection group (*n* = 120). The groups GX005 and S02 were injected with gradient concentrations of 1 × 10^4^, 1 × 10^5^, 1 × 10^6^, 1 × 10^7^, 1 × 10^8^, and 1 × 10^9^ CFUs/mL at 0.2 mL/fish (*n* = 20) of GX005 and S02, respectively. The control group was injected with 0.2 mL of sterile phosphate-buffered saline (PBS) (*n* = 20). The experimental fish were observed daily and fed twice a day for 21 consecutive days.

### 2.4 Safe detoxification test of S02

To investigate the safe detoxification of attenuated S02 vaccine in tilapia, experimental fish of 62 ± 0.81 g were selected. In this experiment, 300 fish were used. The S02 immune group was divided into 50 groups, numbered 1–50 (*n* = 6). S02 was cultivated to 1.0 × 10^9^ CFUs/mL, and an intraperitoneal injection was administrated at a dose of 0.2 mL per fish. Each fish in the No. 1 group was immunized with 0.2 mL of S02. After 24 h, the livers of six fish from the S02 immune group were randomly collected and homogenized in a mortar. A 0.2 mL of liver suspension was injected into the fish in the No. 2 group, and the above method was repeated for all the fish until No. 50 group. A total of 50 generations of safe detoxification tests were conducted in tilapia, and the status and clinical symptoms of each group of fish were recorded and observed daily.

### 2.5 Relative protection rate detection of S02

To investigate the immunogenicity of attenuated S02 vaccine in tilapia, experimental fish 61 ± 6.32 g were selected. A total of 200 fish were evenly divided into the GX005 infection group (control group) and the S02 immune group (*n* = 100). The S02 immune group was injected intraperitoneally with 1 × 10^8^ CFUs/mL of S02 at 0.2 mL/fish, while the GX005 infection group was injected intraperitoneally with 0.2 mL of sterile PBS. After 12 h, all fish were injected with GX005 (3.0 × 10^8^ CFUs/mL, 0.2 mL/fish). The infected fish were continuously observed for 15 days and fed twice a day.

The relative protection rate RPS is calculated as follows:

RPS = {1 − (immune mortality rate ÷ control mortality rate) × 100%}.

### 2.6 Detection of serum IgM level in tilapia

In order to study the changes of intracellular IgM in tilapia immunized with the S02 strain, experimental fish of 60 ± 5.32 g were selected, which were divided into GX005-infected group and S02-immunized group. The S02-immunized group was injected intraperitoneally with 1 × 10^8^ CFUs/mL of S02 at 0.2 mL/fish, while the GX005 infection group was injected intraperitoneally with 0.2 mL of sterile PBS. On the 7th, 14th, 21st, and 28th day after immunization, the fish were randomly selected from each group and their antibody levels were monitored using enzyme-linked immunosorbent assay (ELISA) (*n* = 3). After anesthesia with benzocaine, blood was collected from the fish vein. S02 was diluted with buffer solution (pH 9.6) to 1.0 × 10^8^ CFUs/mL, and coated with a 96-well ELISA plate (100 μL/well), and incubated at 22°C for 60 min. The ELISA plate with PBST (0.1% Tween-20 in PBS) was washed three times. It was blocked with 1% bovine serum for 2 h and washed with PBST three times. The serum was diluted 100 times and added to the well at 22°C for 2 h. It was washed three times with PBST and the anti-tilapia IgM polyclonal antibody was diluted with PBST in a ratio of 1:1,000 at 100 μL/well at 22°C for 1 h. It was then washed with PBST and sheep anti mouse enzyme-linked secondary antibody (1:1,000) was added at 22°C for 1 h, washed three times, and waited for TMB substrate to develop color. The value was read at 450 nm on the enzyme-linked immunosorbent assay reader. After the tilapia was intraperitoneally injected with *Streptococcus* S02 for 2 weeks, the immunized experimental fish were intraperitoneally injected with GX005, and the concentration of GX005 was 5.0 × 10^7^ CFUs/mL at 0.2 mL/fish. The serum of healthy tilapia was collected. No agglutination was detected by hemagglutination test, and the serum was used as a negative serum.

### 2.7 Screening of S02 dosage

To obtain the optimal immune injection dose, the size of tilapia was 61.96 ± 6.14 g, which was divided into six experimental groups and one blank control group (n = 140). Six experimental groups were injected with S02 at concentrations of 1.0 × 10^4^, 1.0 × 10^5^, 1.0 × 10^6^, 1.0 × 10^7^, 1.0 × 10^8^, and 1.0 × 10^9^ CFUs/mL at a dose of 0.2 mL per fish (*n* = 20). The control group was injected with an equal amount of PBS, and the water temperature was maintained at 28–30°C. On the 15th day after immunization, GX005 was used for abdominal infection, with an infection dose of 3.0 × 10^8^ CFUs/mL (100 times LD_50_) at 0.2 mL/fish. The infected fish were continuously observed for 15 days and were fed twice a day.

### 2.8 S02 effect on fish of different sizes

To study the impact of different sizes of fish on immunization efficacy, 120 fish were randomly divided into three groups: tilapia of sizes 15–30 g, 60–80 g, and 90–110 g (*n* = 40 for each group). All three groups of fish received an intraperitoneal injection of 1.0 × 10^8^ CFUs/mL of S02 at a dose of 0.2 mL per fish. The immune water temperature was maintained at 28–30°C. On the 15th day after immunization, GX005 was used for intraperitoneal infection, with an infection dose of 3.0 × 10^8^ CFUs/mL at 0.2 mL/fish. The infected fish were continuously observed for 15 days and fed twice a day.

### 2.9 The effect of water temperature on the efficacy of S02 vaccine

To determine the immune protection period of the attenuated S02 vaccine, experimental fish with a specification of 66.16 ± 4.11 g were selected. The experiment was randomly divided into three water temperature groups: 26, 30, and 33°C. Each temperature group was randomly divided into a blank control group and an S02 immune group (*n* = 50). The tilapia in S02 immune group were injected with 1.0 × 10^8^ CFUs/mL of S02 at a dose of 0.2 mL/fish. The blank control group was injected with the same dose of sterile PBS. The infected fish were continuously monitored for 15 days and fed twice daily. On the 15th day after immunization, GX005 was used for abdominal infection, injected at 4.0 × 10^8^ CFUs/mL at a dose of 0.2 mL/fish. The infected fish were continuously observed for 15 days and fed twice a day.

### 2.10 Determination of immune protection period

To determine the duration of immune protection conferred by the attenuated S02 vaccine, experimental fish weighing 66.16 ± 4.11 g were selected for the study. On the 15th, 30th, 45th, and 60th day post-immunization with the S02 vaccine, GX005 was administered via abdominal infection to the experimental fish (*n* = 150) at a dose of 4.0 × 10^8^ CFUs/mL (0.2 mL/fish). The infected fish were continuously observed and fed twice a day.

### 2.11 Genomic sequencing and annotation

The genomes of GX005 and S02 were sequenced using single-molecule real-time (SMRT) sequencing method at Shanghai Meiji Biopharmaceutical Technology Co., Ltd. The *de novo* sequencing of the bacterial genomes was determined by the bioinformatics method. According to the integrity of the final genome assembly, bacterial genome de novo sequencing was divided into scan map sequencing and complete map sequencing. The scan map uses the second-generation sequencing platform to construct a fragment with an insertion fragment of ~400 bp for qualified DNA samples and then PE150 (pair end) sequencing was performed. The single-ended sequencing reading length was 150 bp, each sample provided raw data of not less than 100× coverage depth of the genome, and finally multiple genome scaffolds were assembled. The completion map adopts the second-generation/third-generation sequencing method. Each sample provides at least 100× third-generation sequencing data (only 30× for pacbio-HIFI sequencing mode) and 100× sequencing data at the same time to ensure more complete and accurate assembly. The completion map can avoid the loss of small plasmid (<15 kb) information and ensure the formation of complete genome containing plasmids.

### 2.12 Genomic composition prediction

Glimmer 3.0 and GeneMarkS software were used to predict the coding sequences (CDS) in the genome. tRNAscan-SE v2.0 software and Barrnap software were employed to predict the tRNA and rRNA present in the genome. The 16 s database was used to predict the 16 s DNA contained in the genome and compared with the House keeping Gene database to obtain housekeeping gene information.

### 2.13 Gene function

Based on the abundance data obtained from different samples, encompassing functions, genes, or mobile elements, rigorous statistical methods were employed to conduct comparative statistical analyses or hypothesis testing among the microbial communities. These analyses aim to evaluate the differences and significance levels of functional categories, genes, and mobile genetic elements within the communities. To achieve this, we utilized Kyoto Encyclopedia of Genes and Genomes (KEGG) and Clusters of Orthologous Groups (COG) databases to ascertain the number of functional categories, genes, and mobile genetic elements present in the samples, as well as to identify those that exhibit significant differences between the samples. Subsequently, we conducted KEGG enrichment analysis on the homologous genes, utilizing R scripts for the purpose of KEGG PATHWAY enrichment analysis. Fisher’s exact test was employed to perform the enrichment analysis. Additionally, we used anti-SMASH (antibiotics and Secondary Metabolite Analysis Shell) to analyze secondary metabolic gene clusters within the samples. To further understand the pathogenicity and resistance profiles of the pathogens, we analyzed the data using the Pathogen Host Interaction (PHI) database, the Virulence Factors of Pathogenic Bacteria (VFDB), and the Antibiotic Resistance Genes Database (ARDB). These resources allowed us to gain insights into the virulence mechanisms and antibiotic resistance capabilities of the microbial communities under investigation.

### 2.14 Comparative genomic analysis

Collinearity generally refers to the similarity in gene arrangement order between certain regions of chromosomes in different species. In the evolutionary process, factors such as chromosomal recombination and gene transposition contribute to the disruption of gene collinearity. Consequently, the level of collinearity between two species can be used as an indicator of their evolutionary distance. Additionally, collinearity analysis becomes instrumental in elucidating the structural variation of genomes between species during the evolutionary process. (1) Constructing gene families using the genes of GX005 and S02: Blast was used to compare all the genes to eliminate solar redundancy and the gene families were clustered utilizing Hcluster sg software to compare the results. (2) As mentioned earlier, the genome alignment results of the samples using MUMmer and LASTZ revealed SNPs, indels, and SVs. (3) A genome overview was created by Circos to display annotation information.

### 2.15 Statistical analysis

All data were analyzed via SPSS 17.0 (IBM Corp., Armonk, NY) to test for normal distributions. For normally distributed data, one-way analysis of variance (ANOVA) followed by the Tukey’s multiple comparison test was used to analyze significance. Asterisks indicate statistically significant differences based on *p*-values as indicated here: * for *p* < 0.05; ** for *p* < 0.01; *** for *p* < 0.001; and **** for *p* < 0.0001.

## 3 Results

### 3.1 The changes in virulence between S02 and GX005 in tilapia

To demonstrate the changes in the virulence between S02 and GX005 vaccines, both S02 and GX005 were used to infect the tilapia through an intraperitoneal injection (i.p.). As shown in Table 1, no mortality was observed in any of the experimental groups exposed to different concentrations of S02. All experimental groups injected with different concentrations of GX005 exhibited mortality. This result indicated that a notable reduction in virulence of S02 when compared to GX005.

**Table 1:** Comparison of virulence between GX005 and S02 (*n* = 20).

Infection routes	Dose (mL)	Concentration (CFUs/mL)	Mortality (%) (No.D/No.Tb)		
			GX005	S02	Control
		1.0 × 10^10^	100% (40/40)	0.00% (0/40)	0.00% (0/40)
		1.0 × 10^9^	100% (40/40)	0.00% (0/40)	
		1.0 × 10^8^	92.5% (37/40)	0.00% (0/40)	
Intraperitoneal	0.2	1.0 × 10^7^	90% (36/40)	0.00% (0/40)	
		1.0 × 10^6^	80% (34/40)	0.00% (0/40)	
		1.0 × 10^5^	50% (20/40)	0.00% (0/40)	
		1.0 × 10^4^	37.5% (15/40)	0.00% (0/40)	

Only one value in each column means that the conditions of all the experimental groups are the same.

### 3.2 Detection of the safety and reversion virulence of S02

S02 was continuously transmitted within tilapia populations across 50 generations, with no incidence of disease, mortality, or other clinical symptoms observed in tilapia. As illustrated in Table 2, S02 strain had undergone complete attenuation, indicating that its utilization for the development of vaccines targeting tilapia poses a minimal safety risk.

**Table 2:** The Safety and Reversion virulence of S02 (*n* = 6).

Generation	Infection routes	Dose (mL)	Concentration (CFUs/mL)	Death/Total
1–10			1.0 × 10^9^	
10–20			1.0 × 10^9^	
20–30	Intraperitoneal	0.2	1.0 × 10^9^	(0/60)
30–40			1.0 × 10^9^	
40–50			1.0 × 10^9^	

Only one value in each column means that the conditions of all the experimental groups are the same.

### 3.3 S02 reduced the mortality rate of tilapia caused by GX005 infection

**Figure 1:**
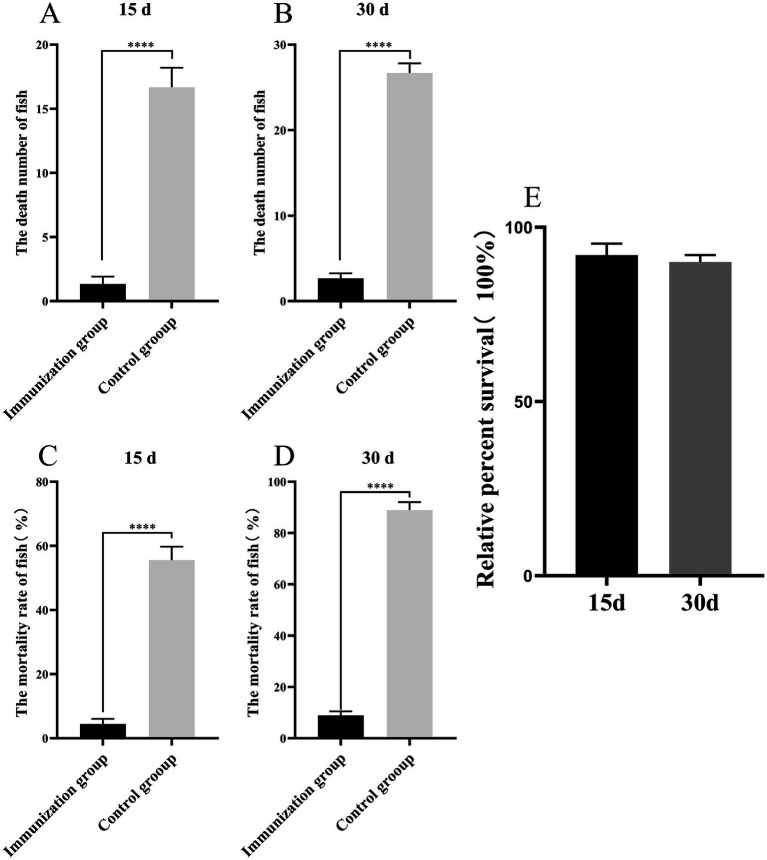
S02 reduces the mortality rate of tilapia caused by GX005 infection (*n* = 100). **(A,B)** The death number of tilapia immunized S02 in 15 and 30 days. **(C,D)** The mortality of tilapia immunized S02 in 15 and 30 days. **(E)** The relative percent survival rates of tilapia immunized with S02 at 15 and 30 days. Statistical analysis was performed by one-way ANOVA followed by Tukey’s multiple comparison test, and expressed as the mean ± SD, ****p < 0.0001 versus the (CX005 infection) Control group.

To evaluate whether S02 vaccination can provide a protective effect against GX005-infected tilapia, we conducted statistical analysis on the number of deaths, mortality rate, and relative protection rate of tilapia immunized for 15 and 30 days. As shown in Figure 1, 15-day post-immunization with S02, the mortality rates in the control and S02-immunized groups were 55.56 and 4.44%, respectively. With regard to 30-day post-immunization with S02, the mortality rates in the control and S02 immunized groups were 88.89 and 8.89%, respectively. The administration of S02 significantly reduced the mortality rate of tilapia, with a relative percent survival (RPS) of 93.25% on the 15th day and 90.31% on the 30th day post-immunization. These results suggested that the attenuated S02 strain as vaccine had the potential to mitigate the mortality of tilapia caused by GX005 infection.

### 3.4 S02 increased the level of IgM in tilapia

**Figure 2:**
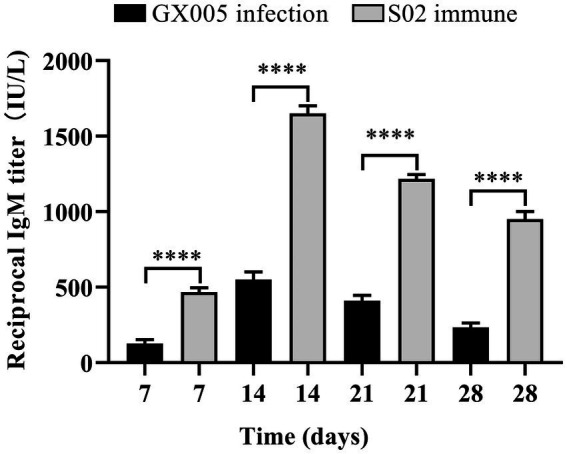
S02 significantly increased the level of IgM in tilapia (*n* = 3). Detection of changes in IgM content in tilapia serum after intraperitoneal injection of immune S02. Statistical analysis was performed by one-way ANOVA followed by Tukey’s multiple comparison test, and expressed as the mean ± SD, ****p < 0.0001 versus the GX005 infection group.

The ELISA results showed that the level of IgM in the serum of tilapia from the S02-immunized group was significantly elevated compared to that in the blank control group (GX005 infection) at the same time point. Furthermore, the level of IgM in the tilapia reached its peak at 14 days post-immunization (Figure 2). These results suggested that immunization with an attenuated S02 strain can significantly increase the level of IgM in tilapia.

### 3.5 Different concentrations of S02 have varying protective effects on tilapia

**Figure 3:**
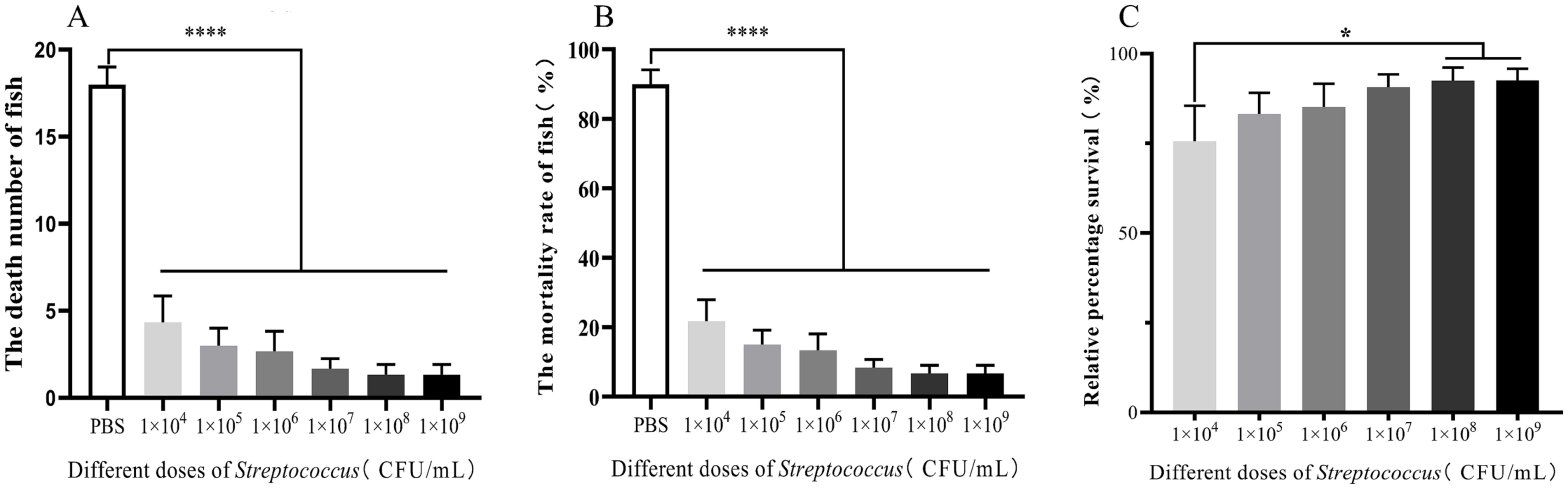
Different dose of S02 have varying protective effects on tilapia (*n* = 20). **(A)** The number of dead fish in tilapia immunized S02 with different doses. **(B)** The mortality of tilapia immunized with S02 at different doses. **(C)** The relative percent survival rates of tilapia immunized with S02 at different doses. Statistical analysis was performed by one-way ANOVA followed by Tukey’s multiple comparison test, and expressed as the mean ± SD, *p < 0.05, ****p < 0.0001 versus the CX005 infection control group.

To investigate the protective effect of different concentrations of the attenuated S02 strain on tilapia, this study designed six groups of gradient immune concentrations for tilapia immunization. On the 15th day post-immunization, the RPS of tilapia immunized with S02 was measured, and the results presented in Figure 3. The graded concentrations of S02 used were 1.0 × 10^4^, 1.0 × 10^5^, 1.0 × 10^6^, 1.0 × 10^7^, 1.0 × 10^8^, and 1.0 × 10^9^ CFUs/mL, respectively, for the six groups. On the 15th day, the RPS values were 75.57, 83.20, 85.16, 90.62, 92.47, and 92.58%, respectively, for these groups. Notably, there was no significant difference in the RPS between the groups immunized with 1.0 × 10^8^ and 1.0 × 10^9^ CFUs/mL at 15 days. The RPS of the group immunized with 1.0 × 10^4^ CFUs/mL was the lowest, significantly differing from the groups immunized with 1.0 × 10^8^ and 1.0 × 10^9^ CFUs/mL. These results indicated that administering 1.0 × 10^8^ and 1.0 × 10^9^ CFUs/mL of S02 had a good protective effect on tilapia.

### 3.6 Study on the effect of S02 immunization on fish of different specifications

**Figure 4:**
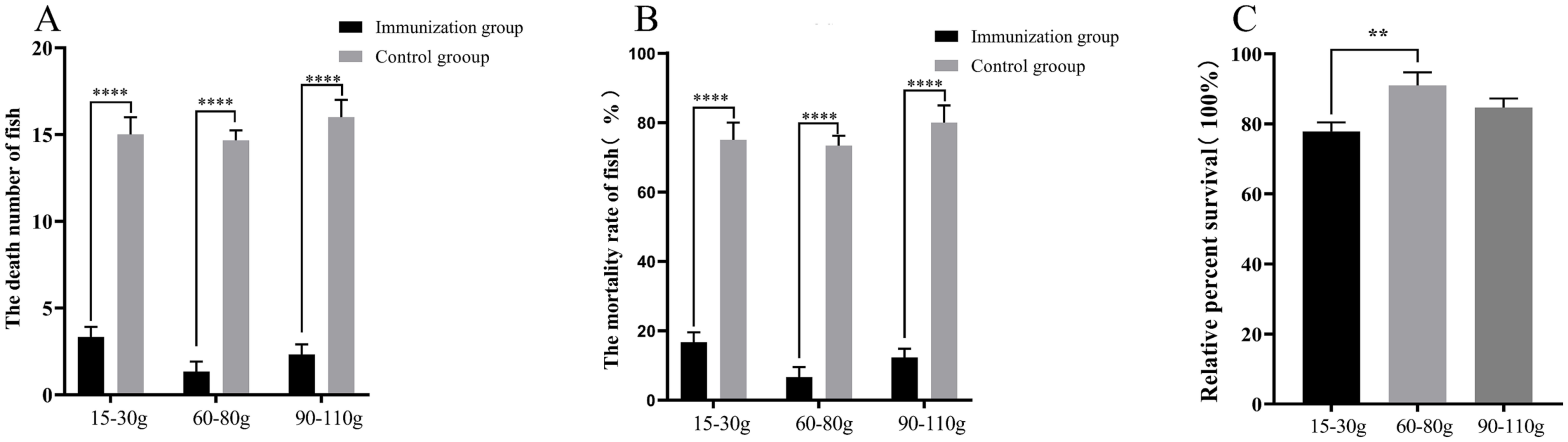
Study of the effect of S02 immunization on fish of different specifications (*n* = 40). **(A)** The number of dead fish in the tilapia of different specifications immunized with S02. **(B)** The mortality of tilapia of different specifications immunized with S02. **(C)** The relative percent survival rates of tilapia of different specifications immunized with S02. **P < 0.01, ****P < 0.0001.

Different fish species exhibit varying degrees of responsiveness to vaccines. To evaluate the protective effect of S02 on different sizes of tilapia, this study selected tilapia weighing 15–30 g, 60–80 g, and 90–110 g for investigation. Immunization with S02 reduced the mortality of tilapia caused by GX005 infection across all the three size groups. The RPS rates of S02 immunization for these three size groups of tilapia were determined to be 77.86, 90.95, and 84.62%, respectively. Notably, the group of tilapia weighing 60–80 g exhibited the highest RPS rate following S02 immunization (Figure 4).

### 3.7 The effect of water temperature on the immune response of S02

**Figure 5:**
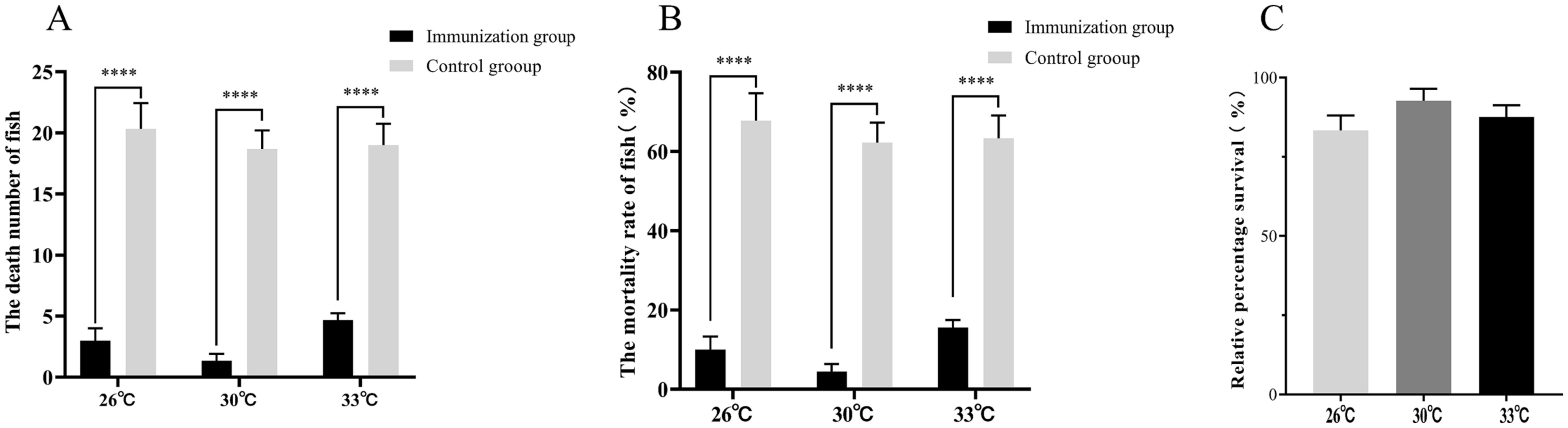
The effect of water temperature on the immune response of S02 (*n* = 150). **(A)** The number of dead fish in the tilapia immunized with S02 at different water temperatures. **(B)** The mortality of tilapia immunized with S02 at different water temperatures. **(C)** The relative percent survival rates of tilapia immunized with S02 with different water temperatures. ****P < 0.0001.

The water temperature has a significant impact on the efficacy of the vaccine. In this study, three different temperatures were chosen: 26, 30, and 33°C. On the 15th day, the RPS rates at these temperatures were 77.86, 90.95, and 84.62%, respectively, (Figure 5). Immunization with S02 at various temperatures demonstrates a notable capacity to mitigate the mortality of tilapia induced by GX005 infection. Notably, the effect is most pronounced at 30°C, albeit without statistically significant differences in RPS among the tested water temperatures.

### 3.8 Determination of immune-protection period

The RPS rate of tilapia on the 15th day was 92.86%, while the relative immune protection rates on the 30th, 45th, and 60th days were 93.99, 91.96, and 90.72%, respectively. The results are shown in Table 3. The duration of immune protection following injection in tilapia extended to 60 days.

**Table 3:** The impact of different immune protection periods on immune efficacy (*n* = 150).

Group	Immunization	Infection time	Concentration (CFUs/mL)	Death/Total	Average mortality (%) rate ± SD	Relative protection rate ± SD
Immunization with S02	S02	15 days	2.0 × 10^8^	7/150	4.67 ± 1.15****	92.86 ± 1.71
Control	PBS	15 days		98/150	65.33 ± 3.06	
Immunization with S02	S02	30 days		6/150	4.00 ± 0****	93.99 ± 0.27
Control	PBS	30 d		100/150	66.67 ± 3.06	
Immunization with S02	S02	45 d		8/150	5.33 ± 1.15****	91.96 ± 1.40
Control	PBS	45 d		99/150	66.00 ± 4.00	
Immunization with S02	S02	60 d		9/150	6.00 ± 2.00****	90.72 ± 2.84
Control	PBS	60 d		97/150	64.67 ± 6.11	

Only one value in each column means that the conditions of all experimental groups are the same. Statistical analysis was performed by one-way ANOVA followed by Tukey’s multiple comparison test and expressed as the mean ± SD, ****P < 0.0001 versus the CX005 infection control group.

### 3.9 Statistical analysis of whole genome sequencing results

Table 4 shows the assembly results of GX005 and S02 strains. The genome size of GX005 is 2,095,003 bp, while the genome size of S02 is 2,036,633 bp. Genomic composition analysis showed that GX005 contains 18 rRNAs, six 16 srRNAs, six 23 srRNAs, six 5srRNAs, and 68 tRNAs. S02 contains 15 rRNAs, five 16 srRNAs, five 23 srRNAs, five 5srRNAs, and 58 tRNAs.

**Table 4:** Statistics of genome components.

	GX005	S02
Gene number	2033	2036
Gene total length (bp)	2,095,003	2,036,633
Gene length/Genome (%)	88.62	88.71
GIs number	18	12
GIs total length (bp)	141,132	155,312
tRNA	68	58
16srRNA	16	16
23srRNA	6	5
5srRNA	6	5
Repbase number	171	180

### 3.10 Geographical indication analysis

**Figure 6:**
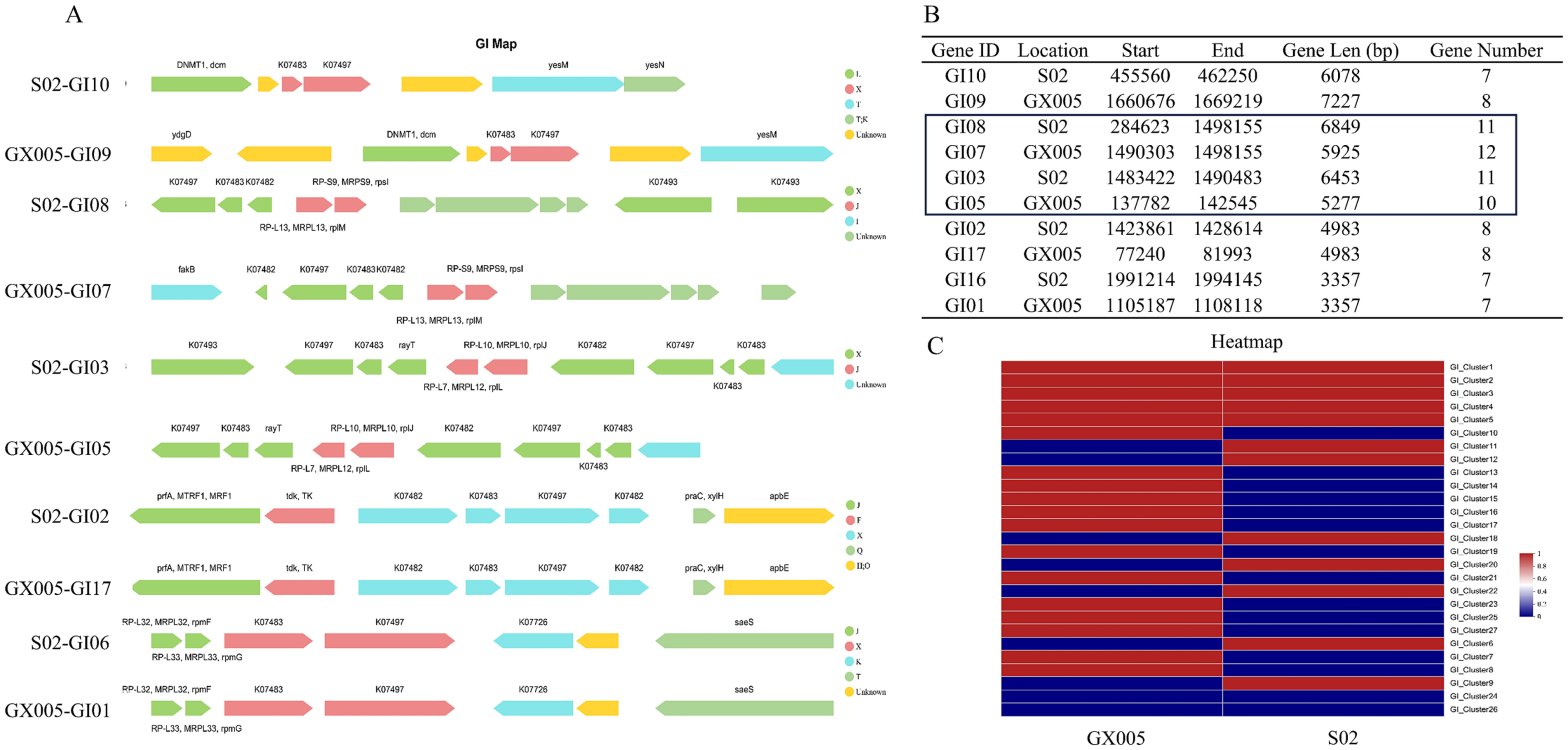
Composition of gene islands (*n* = 6). **(A)** Comparison of gene islands between CX005 and S02 **(B)** Comparison of differential gene islands between CX005 and S02. **(C)** Comparison heatmap of differential gene islands between CX005 and S02. Each row represents a genomic island, with an arrow denoting a gene. The length of the arrow indicates the gene length, while its direction signifies whether the gene is encoded by the sense strand or the antisense strand. The color represents the function of the gene. The letters are the annotation information based on COG (Clusters of Orthologous Groups). F represents nucleotide transport and metabolism; H represents coenzyme transport and metabolism; J represents ribosomal structure and biogenesis; K represents transcription; L represents recombination and repair; O represents post-translational modification; Q represents secondary metabolites biosynthesis; T represents signal transduction mechanisms; X represents nobilome.

Genomic islands (GIs) were predicted based on sequence composition utilizing the IslandPath-DIOMB and Islander software (version 0.2). Strain GX005 harbors 18 GIs, whereas S02 contains 12 GIs, with five GIs being common to both GX005 and S02. There is a significant composition difference between S02-GI08 and GX005-GI07, as well as between S02-GI03 and GX005-GI05 (Figure 6). A comparison analysis of the GIs between GX005 and S02, with a focus on large inversion fragments, revealed that the altered GI segments encode 40 genes in GX005 and 60 genes in S02. Furthermore, GX005 and S02 each have unique sets of 13 and seven genomic islands, respectively.

### 3.11 COG and KEGG pathways analysis in GX005 and S02

**Figure 7:**
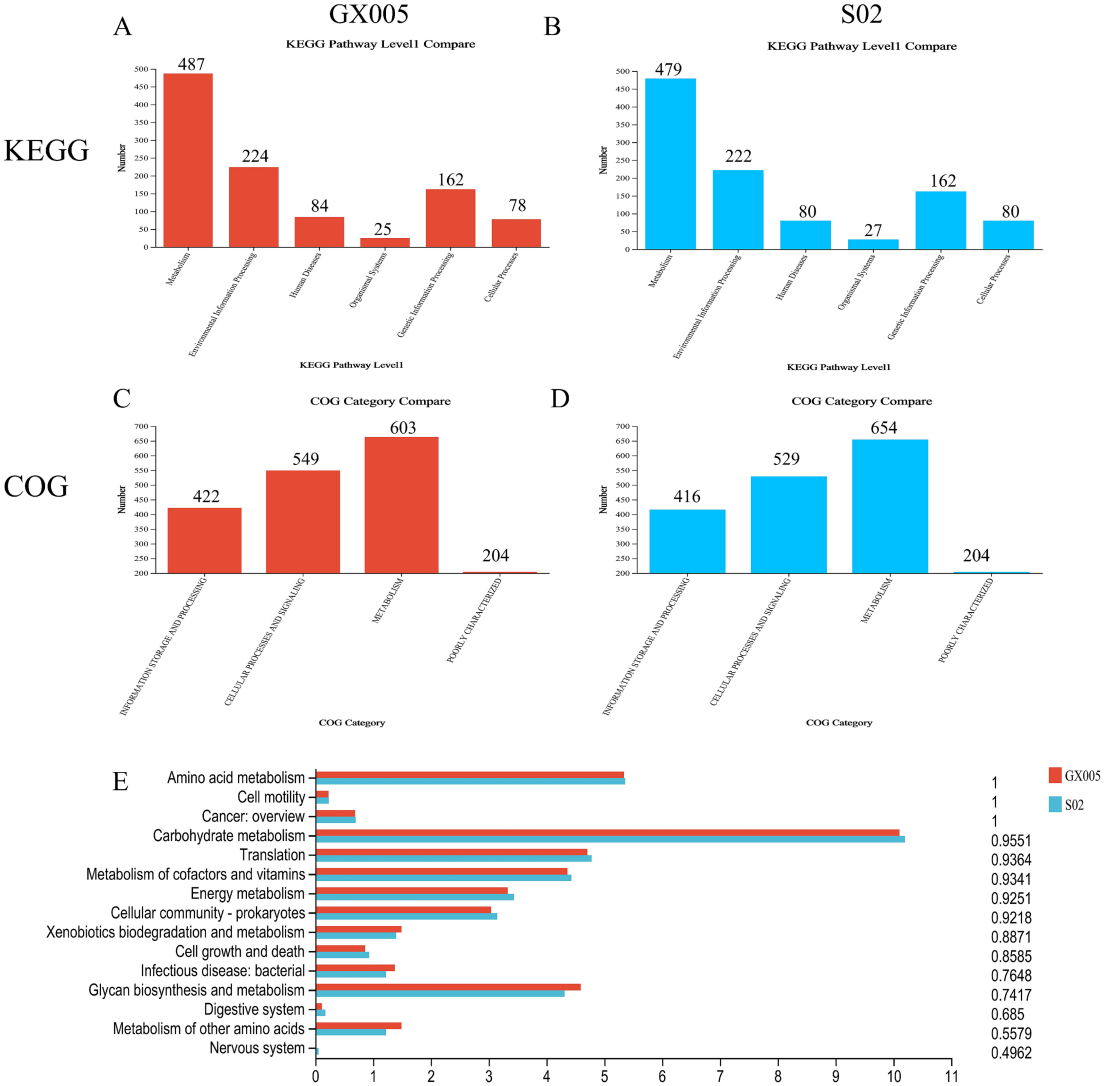
KEGG and COG analysis (*n* = 6). **(A–C)** Comparison of COG analysis between CX005 and S02. **(D,E)** Comparison of KEGG analysis between CX005 and S02.

Functional annotation and COG analysis of the S02 and GX005 genomes were conducted using NCBI non-redundant protein database. No significant differences were observed in GOG analysis between the two strains (Figures 7A–D). To systematically analyze the products and functions, KEGG pathway analysis was performed on GX005 and S02. The results indicated that the primary differences between the strains were in carbohydrate metabolism, glycan biosynthesis and metabolism, and amino acid metabolism (Figure 7C).

### 3.12 Comparison of virulence genes annotated with large reversal fragments

**Figure 8:**
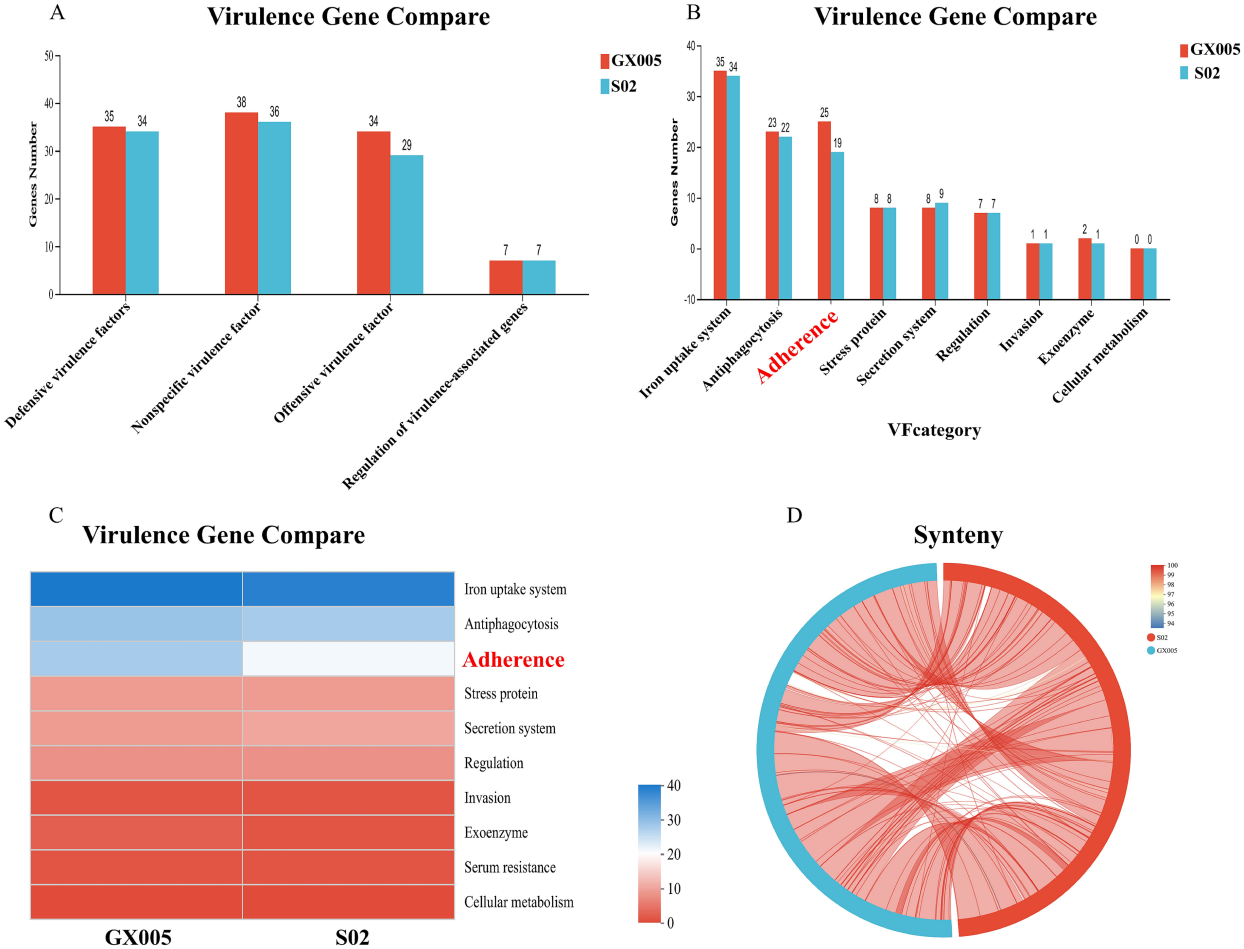
Deletion of hyaluronidase gene and genome collinearity analysis (*n* = 6). **(A)** Comparison of GX005 and S02 virulence genes at level 1. **(B)** Comparison of GX005 and S02 virulence genes at level 2. **(C)** Comparison heatmap of differential virulence genes between CX005 and S02. **(D)** Collinearity diagram of GX005 and S02 virulence genes comparison. **(E)** Circle diagram of differential virulence genes between CX005 and S02.

**Figure 9:**
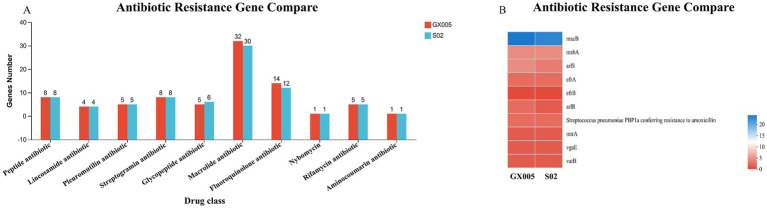
Comprehensive antibiotic resistance database (*n* = 6). **(A)** Comparison of antibiotic resistance genes between CX005 and S02. **(B)** Comparison heatmap of antibiotic resistance gene between CX005 and S02.

Based on the Virulence Factor Database (VFDB), a comparison of the virulence genes in strains GX005 and S02 was conducted (Figure 8). In GX005, the sequence from positions 1,353,484–1,356,987 encodes for hyaluronidase. Conversely, in S02, the deletion of some adherence genes (Figure 8) and hyaluronidase genes (S 1) were noted. Genome collinearity analysis revealed that S02 exhibited inversions of 200,013 base pairs (GX005-350,838–570,838) and 242,630 base pairs (GX005-572,818–570,838–815,448) compared to GX005. The inverted segments encoded 368 genes in GX005 and 372 genes in S02, primarily associated with carbohydrate metabolism, nucleotide metabolism, and amino acid metabolism. Among these, 20 genes were identified as potentially related to virulence (S2) (Figure 9).

## 4 Discussion

*S. iniae* is an important pathogen that infects both aquatic animals and humans, causing significant economic losses (Irion et al., 2021). Current control methods to treat this infection frequently involve the use of antibiotics. However, improper use of these antibiotics can lead to drug residues, contamination of fish products, and an increasing prevalence of antibiotic resistance in bacteria (Mumbo et al., 2023). The evolving resistance of *S. iniae* to antibiotics generates challenges for its control and prevention, while also posing a great threat to public health safety (Xiong et al., 2023). Tilapia is a globally significant species in aquaculture, providing high-quality fish protein to the world. In tilapia farming, infections with *Streptococcus* can significantly affect their growth and economic performance (Khunrang et al., 2023; Eto et al., 2021; Wang et al., 2020). Therefore, there is an urgent need to find better solutions for resisting *Streptococcus* infections beyond the use of antibiotics (Chen et al., 2023). The development of vaccines represents an excellent approach to addressing *Streptococcus* infections, with common immunization methods encompassing injection, immersion, and oral administration. Immunization through injection is typically the most effective, whereas immersion and oral administration offer relatively lower protective efficacy. Attenuated vaccine strains utilized for immunization through injection can more effectively reach the immune organs compared to oral vaccines, exhibiting stable action and immune effects. Consequently, the development of attenuated strains holds great significance for the advancement of injectable vaccines.

Following serial passages, alterations in the morphology of S02 were observed in comparison to GX005, accompanied by a reduction in the size of the hemolytic zone (Supplementary material). The attenuated S02 strain was serially passaged in fish for 50 generations without causing the death of tilapia, which indicated its safety. Injecting a concentration of up to 10^10^ CFUs/mL did not result in the death of tilapia, proving that the strain has lost its pathogenicity to tilapia. The detection of IgM levels showed that the highest IgM levels were observed on the 14th day, consistent with previous reports. Immunizing tilapia with S02 strain resulted in a RPS rate of 93.25% on day 15 and 90.31% on day 30, demonstrating its good immunogenicity. Fish of varying sizes exhibit differential responses to the vaccine (Liu et al., 2016). Among the three sizes tested, tilapia weighing approximately 60 g showed the best immune response, with an immune protection rate of 90.95%. Moreover, the size of the immunized fish, the method of immunization, and temperature, the vaccine concentrations also have an effect on the immune response. This study found that the highest RPS was observed at S02 immune concentrations of 1.0 × 10^8^ CFUs/mL and 1.0 × 10^9^ CFUs/mL (92.47 and 92.58%, respectively). Considering the cost and preparation process in the actual immunization process, a concentration of 10^8^ CFUs/mL can be preferentially selected as the immunization dose. Based on the assessment of immune protection duration and the investigation of IgM levels, supplementary vaccination can be administered according to the IgM concentrations in the serum of the corresponding tilapia. Furthermore, a rational vaccination protocol can be established. Variations in water temperature have a significant impact on fish farming. Fish are highly sensitive to temperature changes (Lusiastuti et al., 2024), which can elicit various stress responses leading to a decline in immunity and an increased risk of bacterial infections (Soto et al., 2014), while also reducing the protective effect of vaccines. Temperature fluctuations affect lipid deposition in the carcass and fatty acid profiles, where lipid accumulation aids fish in resisting stress (de Almeida et al., 2021). Given that fish are poikilothermic animals, the reproductive capacity of S02, as a live vaccine, is directly related to the body temperature of the fish. Literature indicates that the attenuated live vaccine *ALPHA JECT LiVac SRS* can effectively reduce long-term mortality caused by *Pseudomonas salmonicida*, covering approximately the entire lifespan of farmed Atlantic salmon. However, our research also suggests that vaccine efficacy can be compromised by improper use. More specifically, we found that the amount of *P. salmonicida* RNA in the liver after vaccination (representing *in vivo* reproduction of the vaccine) is temperature-dependent, with immunization at 8°C or lower temperatures resulting in a significant loss of efficacy. Our findings are supported by the *in vitro* growth of vaccine strain *AL20542*, which appears to be completely stagnant at these temperatures. Active reproduction of the vaccine strain may be a prerequisite for fully harnessing the potential of attenuated live vaccines (Olsen et al., 2024). Therefore, screening tests that take into account water temperature and fish size can provide a theoretical groundwork for optimizing the protective efficacy of the vaccine. The aforementioned research demonstrated that the obtained attenuated strain has good safety, stability, and immunogenicity, making it a promising vaccine candidate.

By comparing the whole genomes of S02 and GX005, two large inverted fragments and five large gene translocation segments were identified. Functional analysis of these two large inverted fragments revealed that they encode a large number of transposases, endogenous homologous recombination enzymes, site-specific histidine recombinases, and various virulence factors. Studies have reported that large genomic fragment rearrangements occur in bacteria with repetitive sequences and may affect growth and gene expression (Page et al., 2020). Okinaka et al. found that the attenuated phenotype of *Bacillus anthracis* (CDC 684) has large chromosomal inversions that may alter its growth kinetics (Okinaka et al., 2011). During *loxP*-mediated evolution (SCRaMbLE)-induced synthetic chromosomal rearrangements and modifications, genomic deletions, inversions, and translocations occurred in synthetic genomic regions, revealing the potential genetic flexibility of *Candida glabrata* (Ye et al., 2022). Comparative genomic studies of BH15-2 and BH16-24 showed that the main difference lies in a 1.28 Mbp inverted fragment. The inverted fragment may lead to an abnormal expression of drug resistance and virulence genes, which is considered the main reason for the multidrug resistance and reduced virulence of BH16-24. Those researchers found the potential mechanisms underlying the differences in multidrug resistance and virulence among the different genotypes of *S. iniae* (Xiong et al., 2023). Based on their findings, we propose another hypothesis that genomic recombination in the attenuated S02 strain leads to abnormal expression of certain genes within the fragment, ultimately resulting in the reduction of virulence.

The histidine phosphatase family protein is increasingly recognized as an important regulatory post-translational modification in both eukaryotes and prokaryotes (Rigden, 2020). Metal-dependent protein tyrosine phosphatases belonging to the polyphosphate and histidine phosphatase (PHP) family are widespread in Gram-positive bacteria, and protein tyrosine phosphorylation in bacteria plays important roles in a variety of cellular functions, including those related to community development and virulence (Jung et al., 2019). The GNAT family N-acetyltransferase, GCN5-related N-acetyltransferase family (GNAT), is an important protein family that includes more than 100,000 members in both eukaryotes and prokaryotes. Acetylation appears to be a major regulatory post-translational modification, as widespread as phosphorylation (Favrot et al., 2016). Type II toxin–antitoxin (TA) systems are widely distributed in bacterial and archaeal genomes and are involved in a variety of key cellular functions, such as defense against phages, biofilm formation, persistence, and virulence. GNAT toxins have an acetyltransferase activity-dependent translational inhibition mechanism and represent a relatively new and expanding family of type II TA toxins that affect the homeostasis of *Pseudomonas aeruginosa* (Song et al., 2022). Overexpression of 15 GNAT family members in *Streptococcus mutans* demonstrated that acetyltransferase (ActA) impairs its acidogenicity by acetylating LDH, and LDH’s lysine acetylation inhibits its enzymatic activity. Subsequent rat caries model showed that ActA impairs the carcinogenicity of *S. mutans* (Ma et al., 2022). Therefore, gene abnormalities encoding histidine phosphatase family protein and GNAT family N-acetyltransferases may greatly affect bacterial community development, defense against phages, biofilm formation, persistence, and virulence, and reduce the survival rate of bacteria in host cells, leading to a decrease in virulence. Hyaluronidases, produced by a variety of microorganisms including the genes hysA, hylA, and hylB, are capable of degrading hyaluronic acid in connective tissues and initiating the spread of infection by opening the pathways for pathogens to enter host tissues (Hisatsune et al., 2023). Studies have found that hyaluronic acid can modulate the adherence of *Streptococcus suis* to brain microvascular endothelial cells (BMEC), increase the expression of virulence factors in *S. suis*, and enhance the expression of pro-inflammatory cytokines in BMEC (Haas et al., 2015). Compared to the GX005 strain, the S02 strain lacks the hyaluronidase genes hysA, hylA, and hylB, which may be one of the possible reasons for the decreased virulence of S02. Additionally, previous research has shown that GX005_GM000928 is a multidrug ABC transporter permease involved in the transport system of hemolysin located on the large inversion fragment. Hemolysis assays have shown that GX005 has stronger hemolytic activity than S02, supporting the sequencing results. Aberrant expression of these virulence-related genes on the inversion fragment may lead to a decrease in the virulence of GX005. These results need to be further verified to prove the mechanism of virulence change of attenuated SO2 strain.

## 5 Conclusion

In summary, in this study an attenuated strain of *S. iniae*, designated as S02, was chosen as a potential vaccine candidate and a comprehensive investigation into its safety profile, immunogenicity, immune response concentration, and duration of protection was performed. The attenuated S02 strain of *S. iniae* is an excellent candidate as an injectable vaccine. These findings provide theoretical support for the rational application and utilization of the S02 vaccine. Additionally, comparative genomic studies between S02 and its virulent parent strain GX005 indicate that their main differences lie in two 0.2 Mbp inversion fragments, encoding 372 genes, including the GNAT family N-acetyltransferase and hyaluronic acid lyase genes hysA, hylA, and hylB, which are considered the main reasons for the decreased virulence. This study investigated the immune effect and the possible mechanism of virulence attenuation of S02, which provided an excellent candidate strain for the development of vaccines.

## Data Availability

The datasets generated during the current study are available in the NCBI repository. The genome sequences of S. iniae strains GX005 and S02 were deposited into the GenBank under the accession number of PRJNA491220 and PRJNA1134942, respectively.
