# Supplementary material for: Immunoprotective test and whole-genome sequencing analysis of the attenuated S02 strain of Streptococcus iniae
Source: Front Microbiol. 2025 Jun 3;16:1550544. doi: 10.3389/fmicb.2025.1550544 (PMC12170510; doi:10.3389/fmicb.2025.1550544)
Supplement: Supplementary file 1 [file Data_Sheet_1.docx]

Immune protective Test and Whole Genome Sequencing Analysis of the Attenuated Strain S02 of *Streptococcus iniae*

Supplementary materials

**Table 1.** Deletion of hyaluronidase gene.

| Gene Id | Gene Name | Location | VFDB ID | Vfs | Description | Level2 |
| --- | --- | --- | --- | --- | --- | --- |
| gene300006 | ABCF3 | GX005 | VFG000344 | HitABC | iron(III) ABC transporter, ATP-binding protein | Iron uptake system |
| gene300030 | braD, bceA | GX005 | VFG000344 | HitABC | iron(III) ABC transporter, ATP-binding protein | Iron uptake system |
| gene300090 | opuA | GX005 | VFG000344 | HitABC | iron(III) ABC transporter, ATP-binding protein | Iron uptake system |
| gene300125 | potA | GX005 | VFG000344 | HitABC | iron(III) ABC transporter, ATP-binding protein | Iron uptake system |
| gene300190 | yknY, yvrO | GX005 | VFG000344 | HitABC | iron(III) ABC transporter, ATP-binding protein | Iron uptake system |
| gene300323 | ftsE | GX005 | VFG000344 | HitABC | iron(III) ABC transporter, ATP-binding protein | Iron uptake system |
| gene300418 | peb1C, glnQ | GX005 | VFG000344 | HitABC | iron(III) ABC transporter, ATP-binding protein | Iron uptake system |
| gene300604 | metN | GX005 | VFG001206 | FbpABC | iron(III) ABC transporter, ATP-binding protein | Iron uptake system |
| gene300666 | tcyC, tcyN | GX005 | VFG001206 | FbpABC | iron(III) ABC transporter, ATP-binding protein | Iron uptake system |
| gene300711 | ecsA | GX005 | VFG001206 | FbpABC | iron(III) ABC transporter, ATP-binding protein | Iron uptake system |
| gene300779 | htsB, isdF | GX005 | VFG001322 | Isd | iron-regulated surface determinant protein F, ATP-binding-cassette-type transmembrane transporter | Iron uptake system |
| gene300991 | proV | GX005 | VFG001206 | FbpABC | iron(III) ABC transporter, ATP-binding protein | Iron uptake system |
| gene301060 | feoB | GX005 | VFG001859 | FeoAB | ferrous iron transporter B | Iron uptake system |
| gene301111 | ecfA2 | GX005 | VFG001206 | FbpABC | iron(III) ABC transporter, ATP-binding protein | Iron uptake system |
| gene301309 | cydC | GX005 | VFG000366 | Yersiniabactin | inner membrane ABC-transporter YbtQ | Iron uptake system |
| gene301313 | znuC | GX005 | VFG001206 | FbpABC | iron(III) ABC transporter, ATP-binding protein | Iron uptake system |
| gene300769 | endA | GX005 | VFG000970 | DNase | deoxyribonuclease, phage associated | Exoenzyme |
| gene301385 | hysA, hylA, hylB | GX005 | VFG001334 | Hyaluronate lyase | hyaluronidase | Exoenzyme |
| gene301417 | hrtA | GX005 | VFG001206 | FbpABC | iron(III) ABC transporter, ATP-binding protein | Iron uptake system |
| gene301456 | msmX, msmK, malK, sugC, ggtA, msiK | GX005 | VFG000344 | HitABC | iron(III) ABC transporter, ATP-binding protein | Iron uptake system |
| gene301580 | lysY | GX005 | VFG000344 | HitABC | iron(III) ABC transporter, ATP-binding protein | Iron uptake system |
| gene301598 | oppD | GX005 | VFG001206 | FbpABC | iron(III) ABC transporter, ATP-binding protein | Iron uptake system |
| gene301640 | ABC-2. A | GX005 | VFG000344 | HitABC | iron(III) ABC transporter, ATP-binding protein | Iron uptake system |
| gene301682 | afuA, fbpA | GX005 | VFG001205 | FbpABC | iron(III) ABC transporter, periplasmic binding protein | Iron uptake system |
| gene301683 | afuC, fbpC | GX005 | VFG000344 | HitABC | iron(III) ABC transporter, ATP-binding protein | Iron uptake system |
| gene301736 | artR, artM | GX005 | VFG001206 | FbpABC | iron(III) ABC transporter, ATP-binding protein | Iron uptake system |
| gene301884 | glnQ | GX005 | VFG000344 | HitABC | iron(III) ABC transporter, ATP-binding protein | Iron uptake system |
| gene301888 | dltA | GX005 | VFG001403 | Mycobactin | Phenyloxazoline synthase MbtB (phenyloxazoline synthetase) | Iron uptake system |
| gene302038 | ABC.PA.A | GX005 | VFG001206 | FbpABC | iron(III) ABC transporter, ATP-binding protein | Iron uptake system |
| gene302069 | pstB | GX005 | VFG000344 | HitABC | iron(III) ABC transporter, ATP-binding protein | Iron uptake system |
| gene302083 | nupA | GX005 | VFG000344 | HitABC | iron(III) ABC transporter, ATP-binding protein | Iron uptake system |
| gene301370 | znuA | GX005 | VFG001330 | Lmb | zinc transport system substrate-binding protein | Adherence |
| gene301401 | pduQ | GX005 | VFG006717 | Lap | Listeria adhesion protein | Adherence |
| gene301284 | sdrC_D_E | GX005 | VFG001291 | CNA | collagen adhesin precursor | Adherence |
| gene301221 | adhE | GX005 | VFG006717 | Lap | Listeria adhesion protein | Adherence |

**Table 2.** Related virulence genes.

| Location | Start | End | Gene Len (bp) | Gene annotation | Protein ID |
| --- | --- | --- | --- | --- | --- |
| GX005 | 356048 | 359119 | 3071 | "LuxR family transcriptional regulator" | "AYB01209.1" |
| GX005 | 367411 | 368037 | 626 | "sugar ABC transporter substrate-binding protein" | "AYB01217.1" |
| GX005 | 372792 | 374441 | 1649 | "peptide ABC transporter substrate-binding protein" | "AYB01221.1" |
| GX005 | 381320 | 382099 | 779 | "prolipoprotein diacylglyceryl transferase" | "AYB01228.1" |
| GX005 | 382096 | 383028 | 779 | "HPr kinase/phosphorylase" | "AYB01229.1" |
| GX005 | 387549 | 388160 | 611 | "phosphate ABC transporter ATP-binding protein" | "AYB01235.1" |
| GX005 | 388163 | 388978 | 815 | phosphate ABC transporter permease PstA | "AYB01236.1" |
| GX005 | 390731 | 392059 | 1328 | "two-component sensor histidine kinase" | "AYB01239.1" |
| GX005 | 393583 | 394488 | 905 | "permease" | "AYB01243.1" |
| GX005 | 408007 | 408630 | 623 | "ATP-binding cassette domain-containing protein" | "AYB01257.1" |
| GX005 | 408958 | 410133 | 1175 | "IS256 family transposase" | AYB01258.1 |
| GX005 | 421189 | 422019 | 830 | glutamine ABC transporter substrate-binding protein | "AYB01265.1" |
| GX005 | 430809 | 431984 | 1175 | IS256 family transposase | "AYB01276.1" |
| GX005 | 432842 | 433627 | 785 | "multidrug ABC transporter permease" | "AYB01278.1" |
| GX005 | 433629 | 434447 | 818 | "antibiotic ABC transporter permease" | "AYB01279.1" |
| GX005 | 435717 | 436181 | 464 | "IS200/IS605 family transposase" | "AYB01281.1" |
| GX005 | 441165 | 442166 | 1001 | "catabolite control protein A" | "AYB01285.1" |
| GX005 | 448058 | 449233 | 1175 | "IS256 family transposase" | "AYB01293.1" |
| GX005 | 458251 | 459090 | 839 | "Rgg/GadR/MutR family transcriptional regulator" | "AYB01304.1" |
| GX005 | 462132 | 463307 | 1175 | "IS256 family transposase" | "AYB01307.1" |
| GX005 | 448058 | 449233 | 1175 | "IS256 family transposase" | "AYB01293.1" |
| GX005 | 450884 | 451351 | 467 | SsrA-binding protein SmpB | "AYB01296.1" |
| GX005 | 458251 | 459090 | 839 | Rgg/GadR/MutR family transcriptional regulator | AYB01304.1 |
| GX005 | 459367 | 460989 | 1622 | transglutaminase" | "AYB01305.1" |
| GX005 | 462132 | 463307 | 1175 | "IS256 family transposase" | "AYB01307.1" |
| GX005 | 470255 | 471592 | 1337 | "MFS transporter" | "AYB01317.1" |
| GX005 | 473812 | 474348) | 1337 | "peptide-methionine (S)-S-oxide reductase" | AYB01320.1 |
| GX005 | 474414 | 475268 | 854 | "S1 RNA-binding domain-containing protein" | "AYB01321.1" |
| GX005 | 482818 | 483621 | 803 | "peptidylprolyl isomerase" | "AYB01329.1" |
| GX005 | 517915 | 518463 | 548 | "GNAT family N-acetyltransferase" | "AYB01362.1" |
| GX005 | 521703 | 522503 | 800 | signal peptidase II | "AYB01366.1" |
| GX005 | 543024 | 543959 | 935 | "manganese-dependent inorganic pyrophosphatase" | "AYB01388.1" |
| GX005 | 558775 | 559569 | 794 | "glutamate racemase" | "AYB01405.1" |
| GX005 | 567252 | 567743 | 491 | "GNAT family N-acetyltransferase" | "AYB01415.1" |
| GX005 | 617899 | 618255 | 356 | "S1 RNA-binding domain-containing protein" | "AYB01457.1" |
| GX005 | 630694 | 632301 | 1607 | "ribonuclease Y" | "AYB01469.1" |
| GX005 | 646098 | 647012 | 914 | "thioredoxin-disulfide reductase" | "AYB01482.1" |
| GX005 | 657397 | 659031 | 1634 | "ABC transporter permease" | "AYB01492.1" |
| GX005 | 665790 | 666491 | 701 | aquaporin family protein | "AYB01498.1" |
| GX005 | 701458 | 702753 | 1295 | LytR family transcriptional regulator | "AYB01528.1" |
| GX005 | 730833 | 731540 | 707 | "histidine phosphatase family protein" | "AYB01559.1" |
| GX005 | 741668 | 742306 | 638 | "transcriptional regulator" | "AYB01569.1" |
| GX005 | 750415 | 751590 | 1175 | "IS256 family transposase" | "AYB01579.1" |





**Figure 1 Comparison of hemolytic characteristics and gram staining observations.** (A)The comparison of hemolytic characteristics of GX005 and S02. (B) The gram staining observations of GX005 and S02.
